# Mismatch between self-perceived and calculated cardiometabolic disease risk among participants in a prevention program for cardiometabolic disease: a cross-sectional study

**DOI:** 10.1186/s12889-020-08906-z

**Published:** 2020-05-20

**Authors:** D. M. Stol, M. Hollander, O. C. Damman, M. M. J. Nielen, I. F. Badenbroek, F. G. Schellevis, N. J. de Wit

**Affiliations:** 1 Julius Center for Health Sciences and Primary Care, University Medical Center Utrecht, P.O. Box 85500, 3508 GA Utrecht, the Netherlands; 2 Netherlands Institute for Health Services Research (NIVEL), Utrecht, the Netherlands; 3 Amsterdam UMC, Vrije Universiteit Amsterdam, Department of Public and Occupational Health, Amsterdam Public Health research institute, Amsterdam, the Netherlands; 4 Department of General Practice and Elderly Care Medicine, Amsterdam Public Health Research Institute, Amsterdam University Medical Centers (location VUmc), Amsterdam, The Netherlands

**Keywords:** Cardiometabolic diseases, Prevention, Primary care, Risk perception, Risk score, Risk communication, Early detection

## Abstract

**Background:**

The rising prevalence of cardiometabolic diseases (CMD) calls for effective prevention programs. Self-assessment of CMD risk, for example through an online risk score (ORS), might induce risk reducing behavior. However, the concept of disease risk is often difficult for people to understand. Therefore, the study objective was to assess the impact of communicating an individualized CMD risk score through an ORS on perceived risk and to identify risk factors and demographic characteristics associated with risk perception among high-risk participants of a prevention program for CMD.

**Methods:**

A cross-sectional analysis of baseline data from a randomized controlled trial conducted in a primary care setting. Seven thousand five hundred forty-seven individuals aged 45–70 years without recorded CMD, hypertension or hypercholesterolemia participated. The main outcome measures were: 1) differences in cognitive and affective risk perception between the intervention group - who used an ORS and received an individualized CMD risk score- and the control group who answered questions about CMD risk, but did not receive an individualized CMD risk score; 2) risk factors and demographic characteristics associated with risk perception.

**Results:**

No differences were found in cognitive and affective risk perception between the intervention and control group and risk perception was on average low, even among high-risk participants. A positive family history for diabetes type 2 (β0.56, CI95% 0.39–0.73) and cardiovascular disease (β0.28, CI95% 0.13–0.43), BMI ≥25 (β0.27, CI95% 0.12–0.43), high waist circumference (β0.25, CI95% 0.02–0.48) and physical inactivity (β0.30, CI95% 0.16–0.45) were positively associated with cognitive CMD risk perception in high-risk participants. No other risk factors or demographic characteristics were associated with risk perception.

**Conclusions:**

Communicating an individualized CMD risk score did not affect risk perception. A mismatch was found between calculated risk and self-perceived risk in high-risk participants. Family history and BMI seem to affect the level of CMD risk perception more than risk factors such as sex, age and smoking. A dialogue about personal CMD risk between patients and health care professionals might optimize the effect of the provided risk information.

**Trial registration:**

Dutch trial Register number NTR4277, registered 26th Nov 2013.

## Background

The rising prevalence of cardiometabolic diseases (CMD), defined as cardiovascular disease (CVD), diabetes type 2 (DM2) and chronic kidney disease (CKD), calls for effective preventive programs. CVD, DM2 and CKD share risk factors such as dyslipidemia, hypertension, smoking and overweight. Therefore, they are suitable for a combined disease prevention strategy [1]. Self-assessment of CMD risk, for example through an online risk score (ORS) at home, may help to identify individuals at high-risk [1, 2] and might motivate people for risk reducing behavior [3]. For these reasons, an ORS has been incorporated as first step in the Dutch primary care CMD prevention program [2].

Theoretically, ORSs are easy applicable, user friendly, and have the potential to reach many people at risk compared to individual case finding. However, for the effective implementation of an ORS based prevention strategy, it is conditional that individuals understand their risk and perceive it as being of personal relevance [4, 5]. Only then, individuals may engage in risk reducing behavior, such as adopting a healthier lifestyle or visiting a health care professional for advice or treatment [6]. However, it is widely known that for lay people, the concept of ‘personal disease risk’ and the accompanying risk levels and cutoff points are difficult to understand [7]. While health care professionals are familiar with applying mean group results from clinical research to individual cases, for a patient only the individualized risk of disease counts.

Risk perception is a complex concept including not only a cognitive aspect (i.e. the perceived susceptibility to get a disease) but also an affective component (i.e. feelings about the risk, such as worry) [8]. Furthermore, risk perception is influenced by contextual factors such as preexistent beliefs and medical knowledge about risk factors and risk reducing strategies [9–11]. Besides the traditional cognitive aspect, it is also recommended to measure feelings-of-risk that represents the more affective part of risk perception [8, 12, 13].

People who overestimate their CMD risk might have disproportional worries and - as a consequence –unnecessarily consult a health care professional. However – more important – high-risk individuals with low perceptions of their risk might not engage in the necessary lifestyle changes or not consult a health care professional. Qualitative studies have shown a wide variation in the way people use and understand information from ORS [3, 10, 11].

Little is known to what extent the use of an ORS – applied in a primary care setting – actually influences users’ risk perception. In addition, more insight into determinants associated with risk perception in high-risk individuals is needed to optimize future CMD risk communication and management. Therefore, the study objective was to assess the impact of communicating individualized CMD risk scores - by using an ORS - on people’s risk perception and to identify CMD risk factors and demographic characteristics associated with the level of risk perception within high-risk participants of a Dutch prevention program for CMD.

## Method

### Study design

We performed a cross sectional analysis among 7547 participants from the INTEGRATE study, a stepped-wedge randomized controlled trial on the (cost)-effectiveness of a Dutch CMD prevention program in primary care. In 2014 and 2015, 37 participating general practices invited all listed patients aged 45–70 years without an established CMD, hypertension or hypercholesterolemia to participate in a stepwise prevention program for CMD. The ORS was used as a first step in the prevention program to identify high-risk individuals. Details about the design of the INTEGRATE study have been published elsewhere [14]. For the current study, we used baseline data from participants of the intervention and the control group. (see Additional file 1).

### Participants and measurements

#### Intervention group

For the intervention group, we used data of participants who completed the ORS as part of the CMD prevention program. The ORS addressed age, sex, smoking status, body mass index (BMI) (height and weight), waist circumference and family history of DM2 and CVD. Participants immediately received their individualized CMD risk score online (see Additional file 2 and Additional file 3). The ORS was developed to identify high-risk individuals who qualify for further risk examination, including blood pressure measurement and laboratory tests on cholesterol and glucose levels, and was recently externally validated [1, 15]. The threshold for high CMD risk in the ORS was an absolute risk for developing a CMD in the next 7 years of ≥23% for men and ≥ 19% for women [1]. In case of a high-risk for CMD, participants were advised to visit their general practitioner (GP) for further risk profiling. In all other cases they received tailored lifestyle advice and a link to a detailed lifestyle assessment. After completing the ORS, the participants of the intervention group were automatically invited via email to fill out an additional online questionnaire (OQ). The OQ consisted of questions involving demographic characteristics (age, sex, marital status (single; relationship, but not living together; married/living together) and educational level (low: primary and lower secondary education, middle: upper secondary and intermediate vocational education, high: higher vocational education (applied sciences) and university). In addition, the OQ included questions about CMD risk factors: sex, age, smoking (yes/no), BMI (< or ≥ 25 kg/m^2^), waist circumference (≤80 or > 80 cm for women and ≤ 94 or > 94 cm for men), family history of DM2 and CVD (negative/positive), physical activity (active/inactive) and alcohol consumption (≤ or > 14 units per week for women and ≤ or > 21 units per week for men). The cutoff level for physical activity was based on the Dutch recommendation for physical activity which entails 30 min moderate to vigorous exercise per day in 5 days per week [16]. Finally, the OQ comprised questions about risk perception. Individuals’ risk perception of CVD, DM2 and CKD was measured, assessing both cognitive and affective risk perception. Because there is no agreement in the literature on how perceived risk should be optimally assessed, we chose two measures which are known to correlate best with behavioral change [12, 13] Cognitive risk perception was assessed by asking: ‘*how do you estimate you risk for developing 1) cardiovascular disease?’ or* 2) *diabetes?’ or 3) chronic kidney disease?’* Answers were given on a 7-point Likert-scale (1 = extremely low, 7 = extremely high). Affective risk perception was assessed on a 7-point Likert-scale (1 = not worried at all, 7 = extremely worried) by asking: *‘Are you worried about your risk to develop CVD, DM2 and CKD respectively?*

#### Control group

Participants of the control group only filled out the OQ, including the same variables as used in the ORS, so that we were able to calculate their CMD risk, but they neither received an individualized CMD risk score nor a tailored lifestyle advice. One year later these participants were invited for the intervention.

### Analysis

To establish the impact of using the ORS on CMD risk perception we performed a complete case analysis among all participants who had completed the questions about risk perception in the OQ. To create an overall score for CMD risk perception, we calculated composite scores for cognitive and affective risk perception by taking the average of the responses to the risk perception questions regarding CVD, DM2 and CKD. Descriptive statistics were used to present demographics of the intervention and control group (percentages or means). Two-tailed t-tests were used for continuous and Likert-scale outcomes [17] and chi-square test for dichotomous or categorical outcomes to detect differences between the intervention group and control group. Spearman’s correlation was used to correlate calculated risk categories and risk perception scores. Statistically significant differences were defined as a *p*-value < 0.05.

To establish risk factors and demographic characteristics associated with risk perception in case of high-risk, we used data of high-risk participants in the intervention group. All participants in this group had received an individualized risk score and were advised to take “action” (visit the general practice) accordingly.

We built two multivariable linear [17] regression models to establish determinants associated with (cognitive and affective) CMD risk perception. CMD risk perception scores (both cognitive and affective) were used as dependent variables. As independent variables we used sex, age, education level, smoking status, BMI, waist circumference, activity level, alcohol intake and family history for DM2 and CVD. We chose to dichotomize all risk factor variables, because thresholds for a high waist circumference and/or high alcohol intake are different among men and women. Using a continuous scale would have required separate regression models for men and women, resulting in the loss of power. All statistical analyses were performed using STATA version 14.0.

## Results

At the time the analysis was conducted 6400 participants of the intervention group completed the ORS - of which 2172 (34%) completed the ORS and the OQ - in the control group 5375 participants completed the OQ, leaving a study population of 7547 participants. Table 1 shows demographics and risk profiles of the participants. Participants of the intervention group were slightly younger, more often highly educated and less often had a positive family history of DM2 compared to participants in the control group.

**Table 1:** Baseline characteristics, risk factors and CMD risk

	Intervention group	Control group	***P***-value
	*N* = 2172	*N* = 5375	
**Demographics**			
Gender (%)			0.65
Male	45.0	45.6	
Age at randomization (years; mean (SD))	56.1 (SD 7.1)	56.5 (SD 6.9)	< 0.01
Marital status (%)			0.98
Single	14.6	14.6	
Relationship. but not living together	3.3	3.4	
Married/living together	82.1	82.1	
Education level^1^ (%)			< 0.01
Low	12.9	17.6	
Middle	43.3	44.1	
High	43.8	38.3	
**CMD risk factors**			
Positive CVD family history (%)	31.4	33.4	0.09
Positive DM2 family history (%)	19.7	23.1	< 0.01
Current smoker (%)	10.4	14.7	< 0.01
BMI ≥25 (%)	42.0	45.6	< 0.01
High waist circumference^2^ (%)	80.4	81.0	0.51
Physical Inactivity^3^ (%)	48.6	51.7	0.02
High alcohol intake^4^ (%)	14.7	16.1	0.14
**CMD risk**			
CMD^5^ risk based on risk score (%)			0.10
Low	7.0	6.2	
Intermediate	52.7	51.1	
High	40.3	42.8	

Total of percentages may not equal 100% due to rounding

^1^ Education level: low = primary & lower secondary education, middle = upper secondary & intermediate vocational education, high = higher vocational education (applied sciences) & university

^2^ > 80 cm for women and > 94 cm for men

^3^ < 5 days a week of 30 min moderate to vigorous exercise per day

^4^ > 14 units/week for women and > 21 units/week for men

^5^ High absolute risk for men ≥23%, for women ≥19%

Abbreviations: *CVD* cardiovascular disease, *DM2* Diabetes Mellitus type 2, *BMI* body mass index

In addition, more participants of the intervention group showed a healthy lifestyle profile regarding risk factors such as smoking, BMI and physical activity compared to the control group (Table 1). CMD risk based on the online risk score did not significantly differ between groups (40.3% vs. 42.8% had a high-risk).

### Impact of receiving an individualized CMD risk score through an ORS on risk perception

Receiving an individualized CMD risk score did not influence cognitive or affective risk perception.

**Fig. 1:**
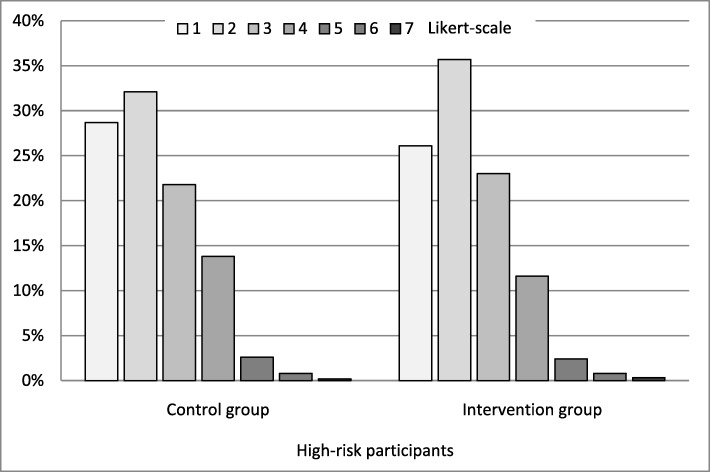
Frequency distribution for cognitive CMD risk perception in high-risk participants

Among high-risk participants, mean cognitive CMD risk perception scores were 2.3 (SD 1.2) in both intervention and control group. For affective risk perception, corresponding scores were 2.0 (SD 1.1) in both groups. Figure 1 shows the frequency distribution on the 7-point Likert-scale for cognitive CMD risk perception in high-risk participants.

In low- and intermediate-risk participants, the scores for cognitive risk perception did not differ either between intervention and control group. In case of low-risk mean cognitive perception scores were 1.6 (SD 0.8) vs. 1.7 (SD 0.9) (*p* = 0.31) and affective perception scores were 1.6 (SD 0.9) vs. 1.5 (SD 0.8) (*p* = 0.07) respectively.

Intermediate-risk participants had mean cognitive risk perception scores of 2.2 (SD 1.1) and mean affective risk perception scores of 2.0 (SD 1.1) in both groups.

In both the intervention and control group, very weak correlations were found between risk categories and cognitive (rho 0.11, *p* = < 0.01 and rho 0.09, *p* = < 0.01, respectively) and affective (rho 0.07, *p* = < 0.01 and rho 0.08, *p* = < 0.01, respectively) risk perception scores.

### Determinants associated with risk perception

Table 2 shows risk factors and demographics associated with cognitive and affective CMD risk perception in high-risk individuals within the intervention group (*n* = 876), who all received a personal risk estimate. A positive family history for DM2 (β 0.56, CI95% 0.39–0.73) and CVD (β 0.28, CI95% 0.13–0.43), a BMI ≥25 (β 0.27, CI95% 0.12–0.43), a high waist circumference (β 0.25, CI95% 0.02–0.48) and inactivity (β 0.30, CI95% 0.16–0.45) were positively associated with cognitive CMD risk perception.

**Table 2:** Multivariable linear regression of demographics and CMD risk factors associated with CMD risk perception among high-risk† participants within the intervention group (*n* = 876)

	Cognitive risk perception			Affective risk perception		
	beta	95% CI	***P***-value	beta	95% CI	***P***-value
Positive DM2 family history	0.56	(0.39–0.73)	< 0.01*	0.42	(0.24–0.59)	< 0.01*
Positive CVD family history	0.28	(0.13–0.43)	< 0.01*	0.15	(−0.00–0.30)	0.06
BMI ≥ 25	0.27	(0.12–0.43)	< 0.01*	0.22	(0.06–0.38)	< 0.01*
Inactivity^1^	0.30	(0.16–0.45)	< 0.01*	0.24	(0.10–0.39)	< 0.01*
High waist circumference^2^	0.25	(0.02–0.48)	0.03*	0.21	(−0.02–0.44)	0.08
Age	−0.01	(−0.03–0.00)	0.08	− 0.01	(− 0.03–0.00)	0.10
Smoking	0.17	(−0.04–0.38)	0.12	− 0.02	(− 0.24–0.19)	0.82
High alcohol intake^3^	− 0.08	(− 0.26–0.10)	0.40	−0.05	(− 0.23–0.14)	0.61
Sex (0 = female, 1 = male)	0.03	(−0.12–0.18)	0.72	0.04	(−0.11–0.19)	0.63
Education level^4^						
middle	0.00	(−0.19–0.19)	0.97	0.00	(−0.19–0.19)	0.99
high	0.01	(−0.19–0.21)	0.92	−0.07	(− 0.27–0.13)	0.46

†High absolute risk for men ≥23%, for women ≥19%

^1^< 5 days a week of 30 min moderate to vigorous exercise per day

^2^> 80 cm for women and > 94 cm for men

^3^> 14 units/week for women and > 21 units/week for men

^4^Education level: low = primary & lower secondary education, middle = upper secondary & intermediate vocational education, high = higher vocational education (applied sciences) & university

*significant

Abbreviations: *CMD* cardiometabolic disease *DM2* Diabetes Mellitus type 2, *CVD* cardiovascular disease, *BMI* body mass index

A positive family history for DM2 (β 0.42, CI95% 0.24–0.59), BMI ≥25 (β 0.22, CI95% 0.06–0.38), and inactivity (β 0.24, CI95% 0.10–0.39) were positively associated with affective risk perception.

## Discussion

### Summary of results

Communicating individualized CMD risk scores by using an ORS had no significant impact on personal risk perception of participants. Risk perception scores in high-risk participants were relatively low in both the intervention and control group, even though the intervention participants had received the result of the ORS. In high-risk participants, a positive family history for DM2 or CVD, BMI ≥25 and physical inactivity were associated with a higher risk perception.

### Interpretation of results

Our finding that receiving an ORS generated individualized CMD risk score did not affect individuals’ risk perception is notable. The underlying assumption of using an ORS, for example as first step in the Dutch CMD prevention program to identify high-risk individuals, is that the ORS helps people to become aware of their risk and initiate preventive actions accordingly. However, even after completing a risk score and receiving personalized CMD risk estimates, most people with a high CMD risk still had low perceptions of risk. Our results confirm the results of Harle and colleagues who found no improvement in risk perception after providing personalized risk estimates through an ORS for DM2 [18]. However the results are in contrast to a recent systematic review [19] which showed that providing patients with CVD risk estimates - primarily oral, written or visual interventions – for primary prevention overall did seem to change risk perception and increased the accuracy of perceived risk. However, the authors indicated that the included studies were heterogeneous (e.g. design and setting) and of low-medium quality. In addition, the included studies rarely assessed web-based interventions.

Several factors have been described which may impede adequate understanding and acceptance of risk.

People seem to associate the readily visible risk factors such as BMI and a positive family history for DM or CVD with CMD risk [20–23] which is supported by our findings. Possible explanations for why these factors influence risk perception are closeness to an affected relative or the experience of his/her illness and the genetic predisposition which makes a positive family history of personal relevance [24–26]. However, risk factors such as age, sex and smoking outweigh the aforementioned risk factors by far in relation to their impact on CMD risk. This discrepancy between perceived risk and calculated risk has also been described in previous studies [20, 27, 28]. Our participants seemed to value CMD risk factors differently than the established epidemiological models.

Apart from existing beliefs about the influence of particular risk factors, other psychological processes can also play a role in processing risk information. For example, motivated skepticism has been reported in the context of receiving breast cancer risk estimates. If the presented risk estimate is different than expected, people tend to question it [29]. In addition, unrealistic optimism about health prospects or defensive coping strategies might also cause rejection of unexpected (high) risk levels [30].

Finally, problems with understanding the communicated risk by an ORS should be taken into account. It is known that people have difficulties with understanding numerical risk [9, 10, 12, 31–33]. Despite the fact that the personalized ORS incorporated important aspects of risk communication - several numerical risk presentations, a visual display of CMD risk, comparative risk information, positive framing and a clear explanation about CMD risk and risk factors – it apparently does not change risk perception.

### Practical implications

A mismatch between calculated risk and risk perception after using an ORS may have major consequences for the effectiveness of CMD prevention programs. Why would patients visit their GP in case of high-risk if they maintain perceptions of low-risk? Interpreting our results, the question rises if an ORS alone is enough to adequately inform people about their risk. Previous qualitative studies have indicated that people prefer to use an ORS together with a health care professional to make sense of the result [5, 34]. To optimize informed decision making, a health care professional could help patients to interpret the result of the ORS, while taking into account their perceptions, preferences and expectations regarding risk management. In addition, the ‘risk-age‘ or ‘lifetime-risk calculator’ could be used to illustrate CMD risk and to show the effects of changing risk factors or lifestyle [35]. Furthermore, raising public awareness about the asymptomatic nature of CMD risk factors and preclinical CMD, the multifactorial etiology of CMD and the multiplicative effect of risk factors could help to improve risk perceptions.

### Strengths and weaknesses

Strengths of this study are the use of a large sample size, its implementation in routine primary care - instead of an evaluation in an experimental setting- and the pragmatic randomized design of the INTEGRATE study, which allowed us to investigate the effect of receiving an online individualized CMD risk score on people’s perceived risk. A second important strength is the fact that we have investigated a web-based intervention.

A number of cautions must also be kept in mind.

First, we performed a cross-sectional analysis on baseline data. Only prospective research can determine whether the associations found exert a causal influence on risk perception. Second, we used only few questions to assess risk perception. However, the risk perception measures were carefully chosen based on previous evidence that these measures predict behavior change best. In addition, it was demonstrated that combining individual risk items into multi-item scales did little to nothing to improve predictions [12]. Moreover, we explicitly wanted to minimize the administrative burden for our participants, especially for those with lower educational levels. Although the questions to measure risk perception were carefully selected and have frequently been used in previous studies [8, 12, 13, 18, 20], these measures were not validated. Potential ramifications are thus an under- or overestimation of the results. However, even if such a measurement error has occurred this would have affected both the intervention and control group, and would not have changed the difference between the two groups. We did not assess people’s absolute risk perception (i.e. a numerical estimate) because there are known difficulties with such an approach [36] and it can be argued that it captures people’s recall of exact numbers rather than how people think or feel about their risk [12, 13] Third, only 34% of the intervention group completed the additional OQ with questions about risk perception. This step was voluntary and may have induced selection bias. The non-responders on the OQ were younger (mean 55.0 vs. 56.1 years; *p* <  0.01) and were more frequently smokers (15.8% vs. 10.4%; *p* <  0.01) compared to the participants. However, a recent study solely among smokers showed that also among this group 62% of the high-risk participants underestimated their CVD risk [37]. Although the OQ was automatically sent after completing the ORS, few participants indicated to have made an appointment with the general practice. Due to the very short time frame, only a handful could have received additional measurements in the meantime. This was supported by the finding that risk perception scores between those who indicated to have made an appointment and those who did not were equal. Fourth, participants of the intervention group were slightly higher educated than the control group. As a result, we might have expected a more accurate risk perception in the intervention group. However, the results did not show such an effect. Finally, the control group seemed to be less healthy than the intervention group concerning certain behavioral risk factors. However, it is important to state that these differences were too small to translate in differences in absolute CMD risk between the groups according to the calculated risk score. Therefore, we assume that these two groups still were fairly comparable. Overall, we believe that these limitations did not vitiate the main conclusions of this paper.

## Conclusion

Communicating individualized CMD risk scores by using an ORS - as part of a CMD prevention program - does not affect individuals’ CMD risk perception. In addition, our results demonstrate a considerable mismatch between calculated CMD risk and individual risk perception. The majority of participants who were informed about a high CMD risk still perceived their risk as being low. A positive family history for DM2 and CVD and a BMI ≥25 seemed to determine individuals’ risk perception more than sex, age and smoking. From our results we conclude that people value CMD risk factors differently than epidemiological models do. A dialogue about personal CMD risk and risk perception between patients and health care professionals seems necessary to optimize the effect of the provided risk information.

## Supplementary information

**Additional file 1.** Flowchart of participants. The online risk score and online questionnaires were filled out at baseline.

**Additional file 2.** Example of risk score for a 62-years old male with a high-risk for CMD. Individuals’ risk is presented as a percentage, a natural frequency (e.g. 68 out of 100 will develop CMD in the next 7 years), a bar chart (including comparison to a peer without risk factors) and a verbal label (e.g. a ‘high risk’).

**Additional file 3.** Example of (non)-contributing risk factors for a 62-years old male with a high-risk for CMD. A list of individuals’ risk factors that contribute to the personalized risk is displayed. On request – by clicking the button- additional information on CMD risk and risk factors is provided. Abbreviations: BMI = body mass index, WC = waist circumference, CVD = cardiovascular disease, DM2 = Diabetes Mellitus type 2.

## Data Availability

The datasets used and/or analysed during the current study are available from the corresponding author on reasonable request.

## Abbreviations

CMD: Cardiometabolic disease
ORS: Online risk score
BMI: Body mass index
CVD: Cardiovascular disease
DM2: Diabetes type 2
CKD: Chronic kidney disease
GP: General practitioner
OQ: Online questionnaire
