# Supplementary material for: Mismatch between self-perceived and calculated cardiometabolic disease risk among participants in a prevention program for cardiometabolic disease: a cross-sectional study
Source: BMC Public Health. 2020 May 20;20:740. doi: 10.1186/s12889-020-08906-z (PMC7238643; doi:10.1186/s12889-020-08906-z)
Supplement: Supplementary file 1 — Additional file 1. Flowchart of participants. The online risk score and online questionnaires were filled out at baseline. [file 12889_2020_8906_MOESM1_ESM.docx]

Online risk score & online questionnaire (complete case)
**n=2,172 (34%)**

Intervention group
**n=16,389**

Control group
**n=14,545**

Online risk score
**n=6,400 (39%)**

Online questionnaire (complete case)
**n=5,375 (37%)**

Invited for INTEGRATE
**n=30,934**
