# Supplementary figures and images for: Mismatch between self-perceived and calculated cardiometabolic disease risk among participants in a prevention program for cardiometabolic disease: a cross-sectional study
Source: BMC Public Health. 2020 May 20;20:740. doi: 10.1186/s12889-020-08906-z (PMC7238643; doi:10.1186/s12889-020-08906-z)

**
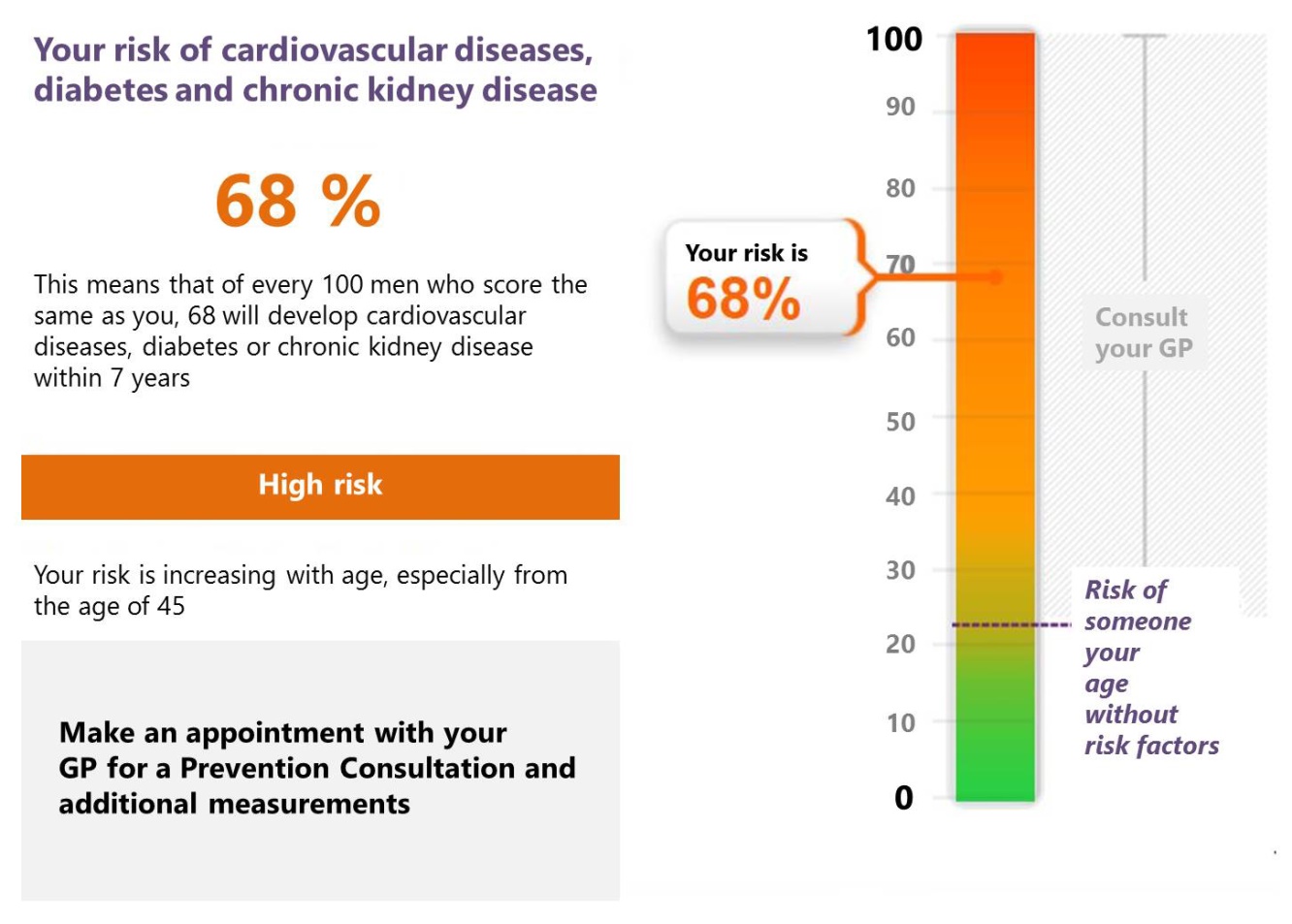
**

Supplement: Supplementary file 2 — Additional file 2. Example of risk score for a 62-years old male with a high-risk for CMD. Individuals’ risk is presented as a percentage, a natural frequency (e.g. 68 out of 100 will develop CMD in the next 7 years), a bar chart (including comparison to a peer without risk factors) and a verbal label (e.g. a ‘high risk’). [file 12889_2020_8906_MOESM2_ESM.docx]

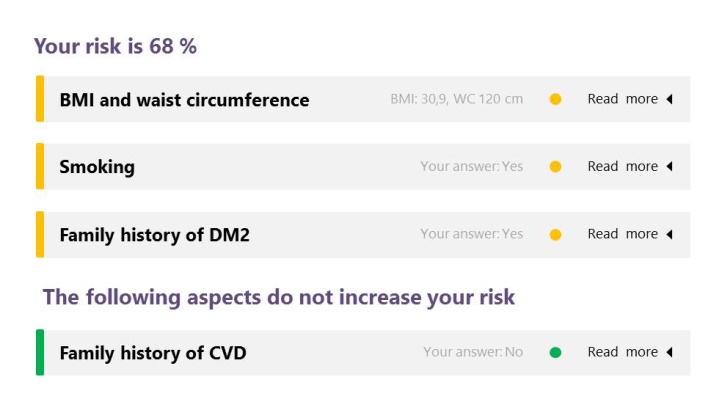

Supplement: Supplementary file 3 — Additional file 3. Example of (non)-contributing risk factors for a 62-years old male with a high-risk for CMD. A list of individuals’ risk factors that contribute to the personalized risk is displayed. On request – by clicking the button- additional information on CMD risk and risk factors is provided. Abbreviations: BMI = body mass index, WC = waist circumference, CVD = cardiovascular disease, DM2 = Diabetes Mellitus type 2. [file 12889_2020_8906_MOESM3_ESM.docx]
